# Potential Molecular Players of the Tumor Microenvironment in Extracranial Pediatric Solid Tumors

**DOI:** 10.3390/cancers12102905

**Published:** 2020-10-09

**Authors:** Rosa Noguera, Tomás Álvaro Naranjo

**Affiliations:** 1 Department of Pathology, Medical School, University of Valencia–INCLIVA Biomedical Health Research Institute, 46010 Valencia, Spain; rosa.noguera@uv.es; 2 Low Prevalence Tumors, Center for Biomedical Research in Cancer Network (CIBERONC), Carlos III Health Institute, 28029 Madrid, Spain; 3 Department of Pathology, Verge de la Cinta Hospital of Tortosa, Catalan Institute of Health, Institut d’Investigació Sanitària Pere Virgili (IISPV), 43500 Tortosa, Spain; 4 Department of Morphological Science, Medical School, Rovira i Virgili University, 43201 Reus, Spain

## 1. Extracranial Pediatric Solid Tumors 

Pediatric cancers are rare malignancies worldwide and represent around 1% of all new cancer diagnoses [1]. Extracranial pediatric solid tumors are a group of nonhematologic, non-central nervous system tumors that occur during childhood. This heterogeneous group of tumors represent approximately 40% of all pediatric cancers [2]. Many pediatric solid tumors are referred to as embryonal or developmental cancers because they arise in young children or adolescents as a result of alterations in the processes of organogenesis or normal growth [3]. However, extracranial pediatric solid tumors are histologically varied and include carcinomas from epithelial cells, embryonal tumors from developing tissues, sex cord gonadal stromal tumors, lymphomas from hematopoietic cells, and sarcomas from mesenchymal cells. The most common extracranial pediatric solid tumors are neuroblastomas, nephroblastomas, lymphomas and rhabdomyosarcomas, each tumor entity exhibiting diverse clinical and biological characteristics [4].

The genomic background of extracranial pediatric solid tumors differs from adult tumors in the low mutational burden and relatively few significantly mutated genes [5]. Certain tumors occurring primarily in children and young adults are marked by characteristic alterations, such as rearrangements upstream to the telomerase reverse transcriptase (TERT) promoter and anaplastic lymphoma kinase (ALK) alterations in neuroblastoma, paired box proteins 3 or 7 (PAX3 or PAX7) fusion in rhabdomyosarcoma tumors and Ewing sarcoma breakpoint region 1 (EWSR1) fusions in Ewing sarcoma [6]. In extracranial pediatric solid tumors, comprehensive molecular profiling studies have detected potentially actionable targets in 46–60.9% of patients [7]. In some cases, identifying genomic alterations has guided targeted therapy selection [8]. Most pediatric malignancies are treated as part of cooperative treatment-optimizing trials based on national and international collaboration. Advances in detection and treatment of childhood cancers have resulted in improved survival for many subtypes, and low clinical stage pediatric solid tumors have relatively successful outcomes compared to the poor outcomes observed in advanced stages, in the presence of metastases or in cases of recurrent disease [9]. Nonetheless, novel therapeutic strategies are needed to improve survival and save patients from the long-term side effects of toxic treatments [10].

## 2. Tumor Microenvironment 

A tumor is a functional and interconnected tissue where malignant cells proliferate uncontrollably, being dependent on their tumor niche or microenvironment. The microenvironment of tumors is a complex network, whereby the fate of a tumor cell is decided not only by its genes, but also by a set of components, including cellular and non-cellular elements, which may have protumoral or antitumoral effects [11]. Among other connections, tumor cells make physical contact through receptor–ligand interactions with surrounding elements, which orchestrate tumor behavior, resulting in a mechanical balance between compression and tension forces to maintain framework architecture stability [12]. This process, known as biotensegrity, is based on the tensegrity principle. A dynamic biotensegral system enables cells and microenvironment to interact, which modifies their structure, especially through integrin–cytoskeleton–nuclear matrix elements [13]. According to this principle, the interplay between tumor cells and extracellular matrix elements can modify cellular and molecular functions, triggering a rigid tumor scaffold; this can result in a variety of changes impacting on global cellular behavior and overall disease progression such as cell proliferation, adhesion, increase in extracellular matrix element deposition, variation of nuclear morphology and genomic integrity, activation of neovascularization, and hypoxia [14]. The functional and structural metabolic aspects of the tumor microenvironment ecosystem also provide clues on which to base new therapeutic approaches, aimed less at destroying cellular and tissue elements, and more at dialogue with them through the processes of proliferation, differentiation, apoptosis, autophagy and energy blockade functioning [15]. Metabolic reprogramming of stromal cells, oxygenation conditions and the pH of the tumor microenvironment provide effective windows of communication with parenchymal tumor cells, which have also reprogrammed their own metabolic condition, their mitochondrial function and inter- and extracellular matrix communication, morphology, capacity for movement and tissue infiltration [16]. Given the biological complexity of extracranial pediatric solid tumors, knowledge of biotensegrity, mechanotransduction, architecture, and topology of its elements, as well as their metabolic interaction, are being increasingly considered [17,18,19]. 

## 3. Future Directions 

Our Special Issue shifts focus away from tumor cells alone, towards considering tumors as complex and dynamic microenvironments with interactions between cells, fibers, vascular structures, and various molecules, with the aim of using them as therapeutic targets. In children, a comprehensive view of their disease is needed, taking into account the tumor macroenvironment (various circumstances of patients and their environment) and following the previously outlined microenvironmental lines, to elucidate factors that improve pre-treatment risk stratification and/or that could be validated as possible therapeutic targets.

Evaluating the combined application of morphometric, topological and genetic strategies in human tumors and in vitro and in vivo systems to determine the contact points of the tumor cell with its extracellular matrix and metabolic and tumor phenotype reprograming in extracranial pediatric solid tumors, our general assumption is that there is an urgent need to establish preclinical models to shine a light on their potential future use as new therapeutic targets. 
